# Formation of Stylet Sheaths *in āere* (in air) from Eight Species of Phytophagous Hemipterans from Six Families (Suborders: Auchenorrhyncha and Sternorrhyncha)

**DOI:** 10.1371/journal.pone.0062444

**Published:** 2013-04-24

**Authors:** J. Kent Morgan, Gary A. Luzio, El-Desouky Ammar, Wayne B. Hunter, David G. Hall, Robert G. Shatters Jr

**Affiliations:** United States Horticultural Research Laboratory, United States Department of Agriculture Agricultural Research Services, Fort Pierce, Florida, United States of America; Volcani Center, Israel

## Abstract

Stylet sheath formation is a common feature among phytophagous hemipterans. These sheaths are considered essential to promote a successful feeding event. Stylet sheath compositions are largely unknown and their mode of solidification remains to be elucidated. This report demonstrates the formation and solidification of *in āere* (in air) produced stylet sheaths by six hemipteran families: *Diaphorina citri* (Psyllidae, Asian citrus psyllid), *Aphis nerii* (Aphididae, oleander/milkweed aphid), *Toxoptera citricida* (Aphididae, brown citrus aphid), *Aphis gossypii* (Aphididae, cotton melon aphid), *Bemisia tabaci* biotype B (Aleyrodidae, whitefly), *Homalodisca vitripennis* (Cicadellidae, glassy-winged sharpshooter), *Ferrisia virgata* (Pseudococcidae, striped mealybug), and *Protopulvinaria pyriformis* (Coccidae, pyriform scale). Examination of *in āere* produced stylet sheaths by confocal and scanning electron microscopy shows a common morphology of an initial flange laid down on the surface of the membrane followed by continuous hollow core structures with sequentially stacked hardened bulbous droplets. Single and multi-branched sheaths were common, whereas mealybug and scale insects typically produced multi-branched sheaths. Micrographs of the *in āere* formed flanges indicate flange sealing upon stylet bundle extraction in *D. citri* and the aphids, while the *B. tabaci* whitefly and *H. vitripennis* glassy-winged sharpshooter flanges remain unsealed. Structural similarity of *in āere* sheaths are apparent in stylet sheaths formed *in planta*, in artificial diets, or in water. The use of ‘Solvy’, a dissolvable membrane, for intact stylet sheath isolation is reported. These observations illustrate for the first time this mode of stylet sheath synthesis adding to the understanding of stylet sheath formation in phytophagous hemipterans and providing tools for future use in structural and compositional analysis.

## Introduction

Many phytophagous hemipterans (true bugs) are characterized by common structural mouthparts [1] that penetrate host plants inter- or intra-cellularly to feed on contents of vascular tissues or other vegetative cell types. The Order Hemiptera is divided into four clade groups (suborders), the Auchenorrhyncha, Coleorrhyncha, Heteroptera, and Sternorrhyncha (for a systematic review of Hemiptera - see Forero, 2008, [1]). The Sternorrhyncha [2], [3], [4] including Psyllidae (e.g. psyllids), Aleyrodidae (e.g. whiteflies), Aphididae (e.g. aphids), Pseudococcidae (e.g. mealybugs), and Coccidae (e.g. scales) and the Auchenorrhyncha including Cicadoidea (e.g. cicadas), Membracoidea (e.g. leafhoppers and treehoppers), Fulgoroidea (e.g. planthoppers), and Cercopoidea (e.g. spittlebugs) contain many agronomically important plant pests that are vectors of pathogens causing plant diseases resulting in vast crop losses worldwide [5], [6], [7].

Phytophagous hemipterans feed by penetration of a stylet bundle into plant tissues. A common trait of these insects is the concurrent formation of a solidifying sheath structure (termed stylet sheath) that encapsulates the stylet bundle while they penetrate into the plant tissues. As the stylets penetrate various plant tissues, they secrete liquid droplets that solidify to form a solid hollow tube extending from the leaf surface to the point of feeding within the plant tissue, often terminating in the plants vascular tissue [8]. Watery and gelling (sheath) saliva represent two common forms of salivary secretions that are implicated in stylet sheaths composition and hemipteran feeding [8], [9], [10], [11], [12], [13], [14], [15]. The exact function(s) of the stylet sheath in feeding are not known; however, trait conservation across phytophagous hemipterans [5], implies biological importance. Stylet sheaths are thought to provide stability and directional orientation to the stylets during the piercing process [16], to aid in proper feeding [16], to ‘cloak’ the stylets from host (plant) defense responses [9], and to seal up cell damage caused by stylet probing [17], [18].

Commonly, sheath initiation occurs concurrently with labium contact with host plant surfaces [19]. It has been suggested that solidification of sheath material requires interaction with host (plant) components [20]. Additionally, oxygen interactions have been suggested for proper gelling of the flange (portion on the leaf surface) and the sheath (section within the leaf tissue) [11], [21], with a recent report supporting this hypothesis [10]. Numerous reports using artificial diet systems have allowed the visualization and study of stylet sheaths [9], [19], [22], [23], [24], [25], [26]. In plant tissues, a typical stylet sheath structure includes a flange (exterior) on the leaf surface, followed (interior) by a narrowing (‘neck’ region) as the stylet sheath traverses the upper/lower (outer) epidermis of the leaf [22], [27]. This is subsequently followed by a thicker sheath ‘trunk’ having a continuous sequential bulbous structure as it traverses the tissue to the target vascular region (phloem or xylem) where the sheath may remain as a single channel or branch laterally throughout the vascular tissue, also in a continuous sequential bulbous form [22], [27], [28]. Recently, sheath material was induced using a brushing technique in sharpshooter [29]; however, the shape of this induced solidified sheath material differs from those formed naturally [30], [31].

In this report, we demonstrate the non-induced formation of stylet sheaths ‘in air’ (*in āere*) across a single-layer membrane surface for *Diaphorina citri* (Psyllidae, Asian citrus psyllid) housed in ‘mock’ feeding chambers (MFC) lacking diet. Membrane probing and stylet sheath formation by the *D. citri* was virtually instantaneous upon caging in MFCs. The caged *D. citri* penetrated the single layer membrane with their stylets, depositing fully formed *in āere* stylet sheaths. To find out if this attribute is common in other phytophagous hemipteran families, we similarly tested: *Aphis nerii* (Aphididae, oleander aphid), *Aphis gossypii* (Aphididae, cotton/melon aphid), *Toxoptera citricida* (Aphididae, brown citrus aphid), *Bemisia tabaci* biotype B (Aleyrodidae, whitefly), *Homalodisca vitripennis* (Cicadellidae, glassy-winged sharpshooter), *Ferrisia virgata* (Pseudococcidae, striped mealybug), and *Protopulvinaria pyriformis* (Coccidae, pyriform scale) each producing *in āere* stylet sheaths. Subsequent to this, we developed a technique to isolate intact stylet sheaths utilizing a water-soluble polyvinyl alcohol membrane and differential filtration. We present this method of sheath production as a tool for downstream sheath compositional and structural analyses without complicating interactions of foreign material of either plant or artificial diet origin.

## Materials and Methods

### Insects

We obtained *Diaphorina citri*, *Homalodisca vitripennis*, *Bemisia tabaci* biotype B, *Toxoptera citricida*, and *Ferrisia virgata* from colonies maintained at the USDA-ARS, United States Horticultural Research Laboratory (USHRL) at Fort Pierce, FL. *Aphis nerii* were isolated from milkweed (*Asclepias tuberose*) plants (GPS: N 27 25.730, W 80 24.538), *Aphis gossypii* from hibiscus (*Hibiscus rosa-sinensis*) plants (GPS: N 27 25.696, W 80 24.503), and *Protopulvinaria pyriformis* from Confederate Jasmine (*Trachelospermum jasminoides*) vine (GPS: N 27 25.703, W 80 24.536) located within the USHRL property. Insect identifications were obtained from Dr. Susan Halbert and Dr. Ian Stocks, Florida Department of Agriculture and Consumer Services, Division of Plant Industries, Gainesville, FL.

### Insect Chambers

Insect chambers consisted of single species of insects (see section 2.1) caged individually and/or in groups within standard (100 mm×15 mm) or smaller (35 mm×10 mm) plastic Petri dishes. A single Parafilm® or clear plastic ‘kitchen’ wrap (multiple brands tried, each being viable) layer was stretched across the top of the plate to seal it from insect escape and to provide a stylet penetrable surface for insect probing; hereafter called a ‘mock feeding chamber’ (MFC). Parts of the formed stylet sheaths on the membrane interior (directly accessible to the insect) and exterior (insect stylet probe accessible) surfaces are hereafter designated ‘flange’ and ‘sheath face’, respectively.

MFCs were maintained within clear Pyrex® baking dishes with the membrane (film) side up and the baking dish covered with a single layer of clear plastic wrap to prevent external contaminant interactions with the membrane surface during incubation. MFCs were incubated at temperature (25°C), relative humidity (75%), and light:dark cycle of 14∶10 hours during experiments that ranged from 0–10 minutes up to 72 hours in duration.

### Whole Stylet Sheath Collection

To collect intact *D. citri* stylet sheaths (sheath with flange attached), we used Solvy™ Stabilizer (Sulky®, Kennesaw, GA), a water-soluble polyvinyl alcohol membrane, that provided a stylet penetrable probing surface for sheath deposition. After deposition, the membrane was removed from the MFC and washed with 100% ethanol (Aaper, Shelbyville, KY) to remove insect debris. The membrane was then immersed in Nanopure H_2_O to dissolve and liberate the sheaths into solution. This solution was sequentially filtered using a Nalgene reusable vacuum filtration system (Thermo Scientific, Waltham, MA) with a combination of Spectra/mesh® polyester membranes (Spectrum Labs, Rancho Dominguez, CA) of 50 µm and 10 µm pore sizes. The filter (50 µm) was used to separate large particulates from the sheath/Solvy™ solution and the filtrate was then passed through a 10 µm filter capturing intact stylet sheaths. The filter (10 µm) was then carefully removed from the vacuum filtering system and gently washed with a gentle stream of 100% ethanol to rinse the sheaths from the filter’s surface. The ethanol/stylet sheath rinse was carefully collected into a clean glass container and stored at −4°C for analyses. Spectra/mesh membrane pore sizes were adjusted depending on the size of the expected sheaths for capture/harvest.

### Scanning Electron Microscopy (SEM)

SEM micrographs were obtained using two methods. The first method employed a ‘tape lift’ procedure using 6 mm diameter SEM carbon adhesive tabs (Electron Microscope Sciences [EMS], Hatfield, PA) affixed to the center of SEM aluminum specimen mounts (EMS). Utilizing the stub base as a handle, the exposed adhesive surface was gently affixed and subsequently detached (repeatedly) across the sheath face of a MFC directly attaching air formed sheaths to the carbon adhesive tab surface, resulting in partial sheath isolation (minus the flange). These were then directly analyzed by SEM (pre-sputtering) using a Hitachi S-4800 Scanning Electron Microscope, (Hitachi High-Technologies Corporation, Tokyo, Japan) at 5 KV, 7 KV, 10 KV or 25 KV. Subsequently the stubs were gold/palladium sputter coated using a Gold/Palladium Hummer ™ 6.2 sputtering system (Anatech USA, Union City, CA) then reanalyzed by SEM.

The second method employed a novel technique for direct SEM imagery of both membrane surfaces (flange and sheath faces). Briefly, Cellstar® 35 mm×10 mm tissue culture (Greiner Bio-One North America Inc., Monroe, NC) MFCs using clear plastic wrap membrane were made for each of the insects used in this study. Chambers were incubated for 2 to 24 hours depending on the insect used and its propensity to probe the membrane. Following this, the sidewalls of the MFCs were drilled with two opposing holes using a flame heated 20 gauge needle to puncture through the plastic sidewall allowing the MFCs interior to equilibrate in the vacuum of the gold/palladium sputter coating system. Given the stringency involved with direct surface flange/sheath SEM analysis, plastic wrap provided a greater material stability/endurance during the sputter plating and SEM processes as opposed to the more ductile Parafilm®.

To analyze the flange face (interior membrane surface), the membrane was carefully removed from the MFC of interest and replaced inverted (flange face up) and stretched across a like-sized Petri dish. These new chambers were subsequently drilled, sputter coated, and the flange deposits of selected insects were observed by SEM. With SEM micrograph imagery capture was performed using the FE-PC SEM bundled software included in the SEM microscope control system.

### Transmission Electron Microscopy (TEM)

*D. citri* adults feeding on citrus leaves in propylene tubes [32] were immobilized (while feeding) by placing the rearing tube in a −20°C freezer for 10 minutes. These adults were then gently pulled away from the leaf (with their stylets still extended) using fine forceps under a stereomicroscope. *D. citri* heads with extended stylets were immediately fixed in 2.5% glutaraldehyde in sodium cacodylate buffer (pH 7.4) at 4°C for 3 to 4 days, post fixed in 2% osmium tetroxide in the same buffer for 1 to 2 hours at 4°C, washed in buffer, dehydrated in ethanol and acetonitrile (substitute for acetone), then embedded in EMBed-812 (both from EMS). Ultrathin sections were stained with uranyl acetate and lead citrate and examined with a Hitachi S-4800 electron microscope (in transmission mode) at 25 KV.

### Confocal Microscopy

For confocal differential interference contrast (DIC) and confocal laser microscopy, a Zeiss LSM 510 AXIO Imager M1 confocal microscope was used (Carl Zeiss MicroImaging GmbH, Jena, Germany) controlled by Zen software version 2009 SP2 (Zeiss). To mount the *in āere* stylet sheaths on glass slides, 50 µL of mounting medium (70% glycerol in 30% PBS) was applied directly to the sheath surface of the MFC for direct harvest of partial sheaths from the membrane surface using a bent glass rod. Then 10 µL of this medium (with harvested stylet sheaths) was directly pipetted onto pre-cleaned microscope slides. Glass covers (No. 1 thickness, Fisher Scientific, Hampton, NH) were applied and their edges sealed with clear nail polish (EMS). Solvy™ isolated whole *D. citri* stylet sheaths, were imaged by confocal DIC post stylet sheath collection, mounted on pre-cleaned microscope slides, covered, and sealed with nail polish as described previously. In some cases, *D. citri* nymphal exuviae were gently pulled away from the leaf (with their stylets still extended) under a stereomicroscope, preserved in 70% ethanol, then mounted and examined by confocal DIC as described. Additionally, hand sections of Duncan grapefruit (*Citrus paradisi* Macfadyen) leaf midribs fed on by *D. citri* were stained with propidium iodide following standard protocols and mounted for confocal laser and confocal DIC microscopy as described previously [33].

### Stereoscopic Microscopy

Stereoscopic micrographs and video were acquired using a Leica M60 Stereomicroscope and Leica DFC290 HD camera system with image and movie capture by LAS (Leica Application Suite V3.7.0 build 681, Leica Microsystems, Wetzlar, Germany) imagery software.

### Light Direct Sheath Face Mock Feeding Chamber Microscopy

Direct MFC sheath face microscopy employed the 35 mm×10 mm chambers or 100 mm×15 mm MFCs. For direct sheath face light microscopy, we used an Olympus IX70 inverted microscope (Olympus, Tokyo, Japan). Micrographs were captured using an Olympus DP73 camera system (Olympus) coupled with cellSens® Dimension version 1.6 (build 9457) imaging software (Olympus). The direct examination of the MFC membrane affixed stylet sheaths was accomplished by inverting the MFC, membrane/sheath face down, directly over an inverted microscope objective lens. This allowed for direct visualization of membrane-affixed stylet sheaths using 4, 10, 20, and 40X objectives. The addition of mounting medium and subsequent overlay with a thin glass cover slip directly to the MFC membrane surface, permitted use of 60 and 100X objectives with oil immersion. Illumination of the stylet sheaths employed either in-line (parallel light to the objective) or 90° lighting (perpendicular to the objective, provided by an external halogen light).

Video capture of live action insect mock feeding attempts employed the Sony DKC-5000 camera system (Sony, Tokyo, Japan) coupled with Image Pro Plus 6.0 software (version 6.0.0.260, Media Cybernetics, Inc., Bethesda, Maryland).

### Micrograph and Video Tools

Paint.Net version 3.5.6 (Copyright^©^ 2010 dotPDN LLC, Rick Brewster, and contributors, http://www.getpaint.net/index.html) was used for addition or repositioning of scale bars, size cropping of images, or image rotation as needed. ImageJ version 1.42q (Rasband, W.S., ImageJ, U. S. National Institutes of Health, Bethesda, Maryland, USA, http://imagej.nih.gov/ij/, 1997–2011) was used to render confocal images into 3D projects and for size measurements. Windows Live Movie Maker version 2011 (Microsoft) was used for video editing, and Any Video Converter version 3.5.2 (AVCLabs, www.avclabs.com) was used for video conversion and compression.

## Results

### Formation of *in āere* Stylet Sheaths in Tested Phytophagous Hemipterans

**Figure 1:**
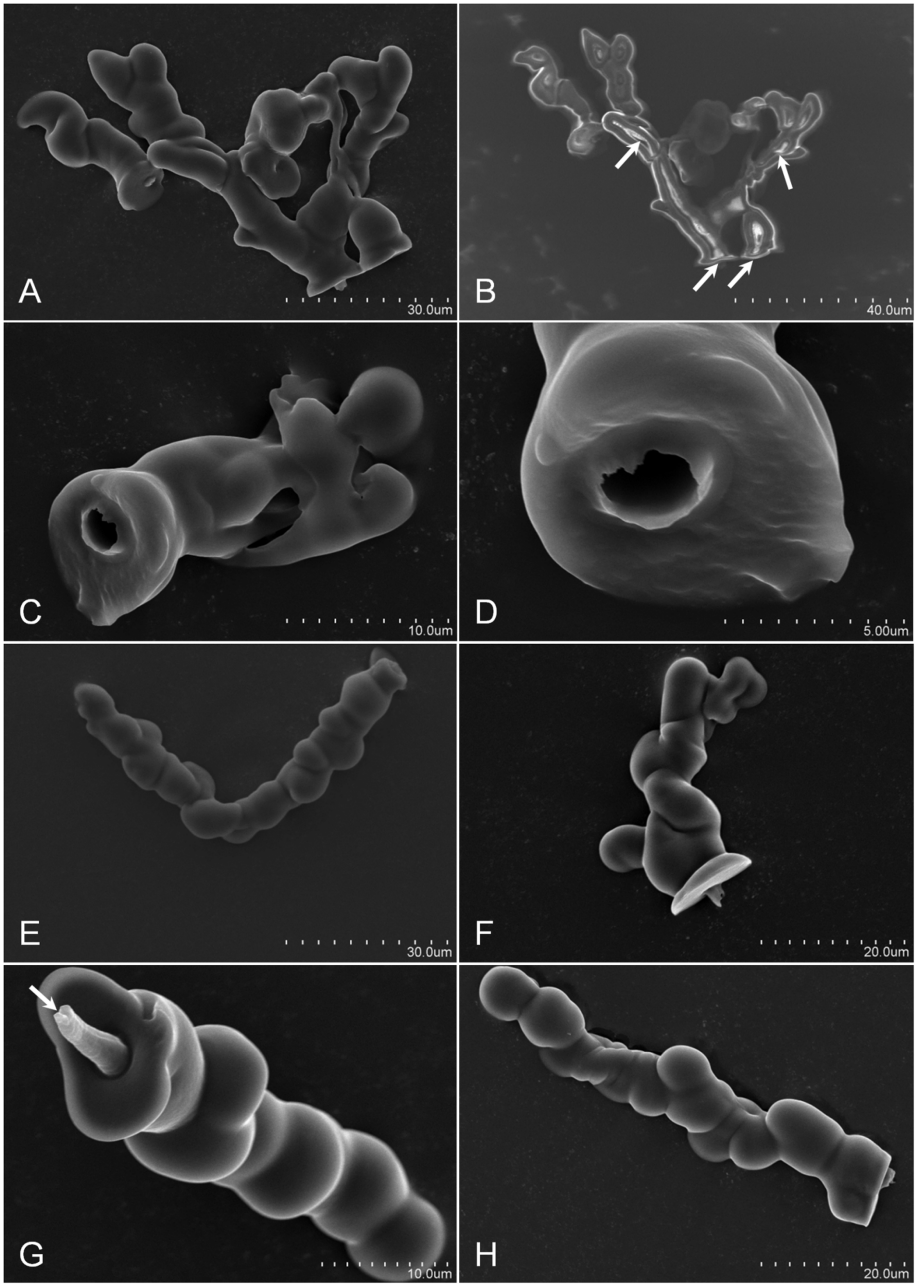
Scanning electron micrographs of *D.citri in a¯ere* stylet sheaths. Panel A, is an image of two sheaths, the first (left sheath) is single-canal and the second (right) is a multi-branched sheath. Panel B is a non-gold sputtered image of Panel A with the white arrows indicating internal hollow canal tracks for the *D. citri* stylets. Panels C and D indicate the hollow canal of the *D. citri* stylets track with Panel D being an enlargement Panel C stylets canal opening. The opening is ∼3 µm in diameter. Panels E and F, indicate angular stylets probing (*in āere*) with panel E having a ∼93° bend within a single-canal sheath. Panels G and H are typical of linear sheaths formed *in āere* with panel G (white arrow indicating a closure of the stylet canal) suggesting secretion of sheath material as the stylet was retracted from the sheath.

**Figure 2:**
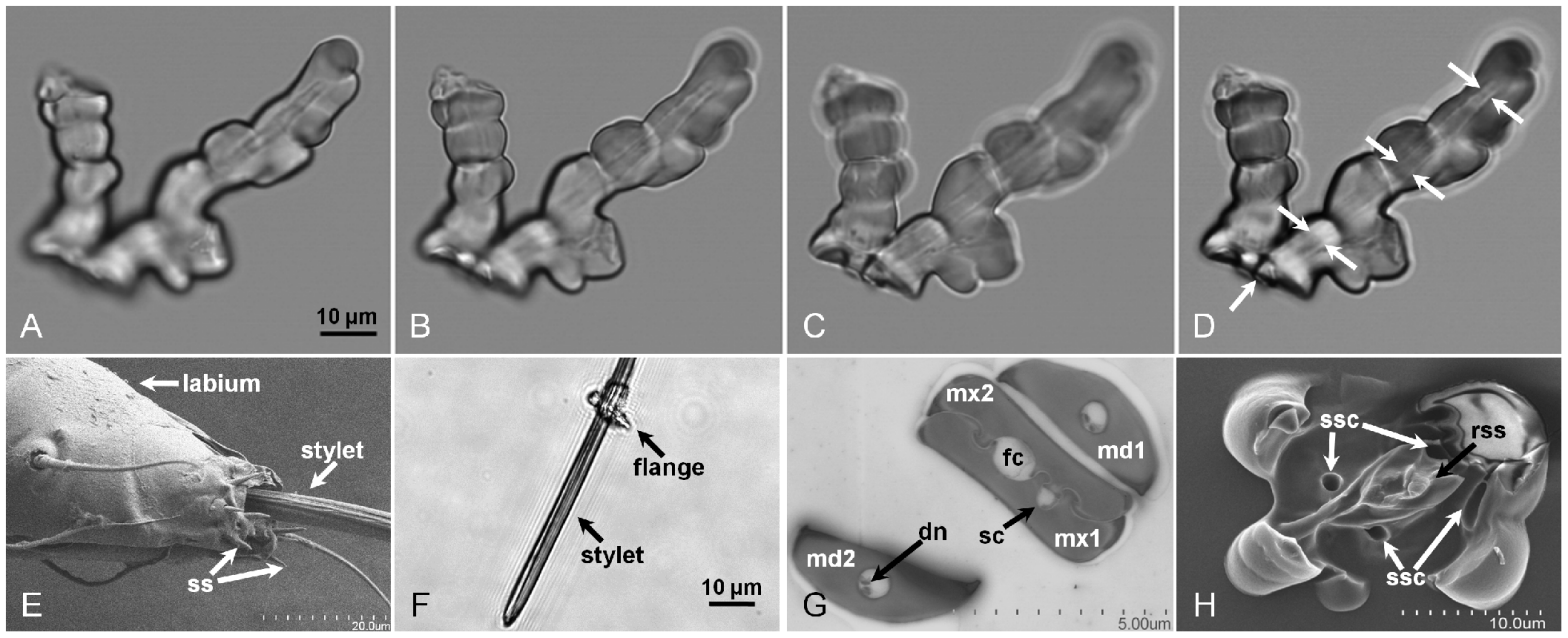
Micrograph images of *D.citri in a¯ere* formed stylet sheath and flange, and *D. citri* labrum and stylet bundle. Panels A – C are individual confocal slices, with Panel D being a merged composite image of Panels A – C confocal slice images. Panel D, white arrows, indicate the visible internal stylet canal traversing the central core of the stylet sheath extending from the base to the terminal end of the sheath. Panel E is a SEM micrograph of a *D. citri* labium, stylets, and labial tip sensilla (ss). Panel F is a single slice confocal DIC micrograph of a *D. citri* third instar nymph exuvial stylet bundle with a detached flange circumambient the stylets. Panel G is a TEM micrograph cross-section of an adult *D. citri* stylet bundle indicating interlocking maxillary (mx) with mandibular (md) stylets, salivary (sc), food (fc), and dendrite (dn) canals. Panel H is a SEM micrograph of an adult *D. citri* flange formed in a mock feeding chamber. White arrows designate the location of indentations in the flange are sensilla cavities (ssc) with the central protrusion (black arrow) indicating a mound of retraction secreted sheath (rss) material formed upon withdrawal of the stylets from the sheath.

During the *D. citri* stylet sheath studies using artificial diet sandwiched between two layers of Parafilm® membrane [34], we observed that immediately upon caging in a Petri dish covered with a single layer of Parafilm® prior to diet overlay, the *D. citri* initiated feeding behavior across the membrane (Video S1). Subsequent to this observation, *D. citri* were caged in MFCs using different membrane types including a variety of clear plastic wrap(s) and Solvy™ stabilizers (water-soluble poly-vinyl alcohol membranes). On all of these membranes, the *D. citri* produced stylet sheaths *in āere* (e.g. Figure 1; Figure 2A–D; and Figure S1G–I).

**Figure 3:**
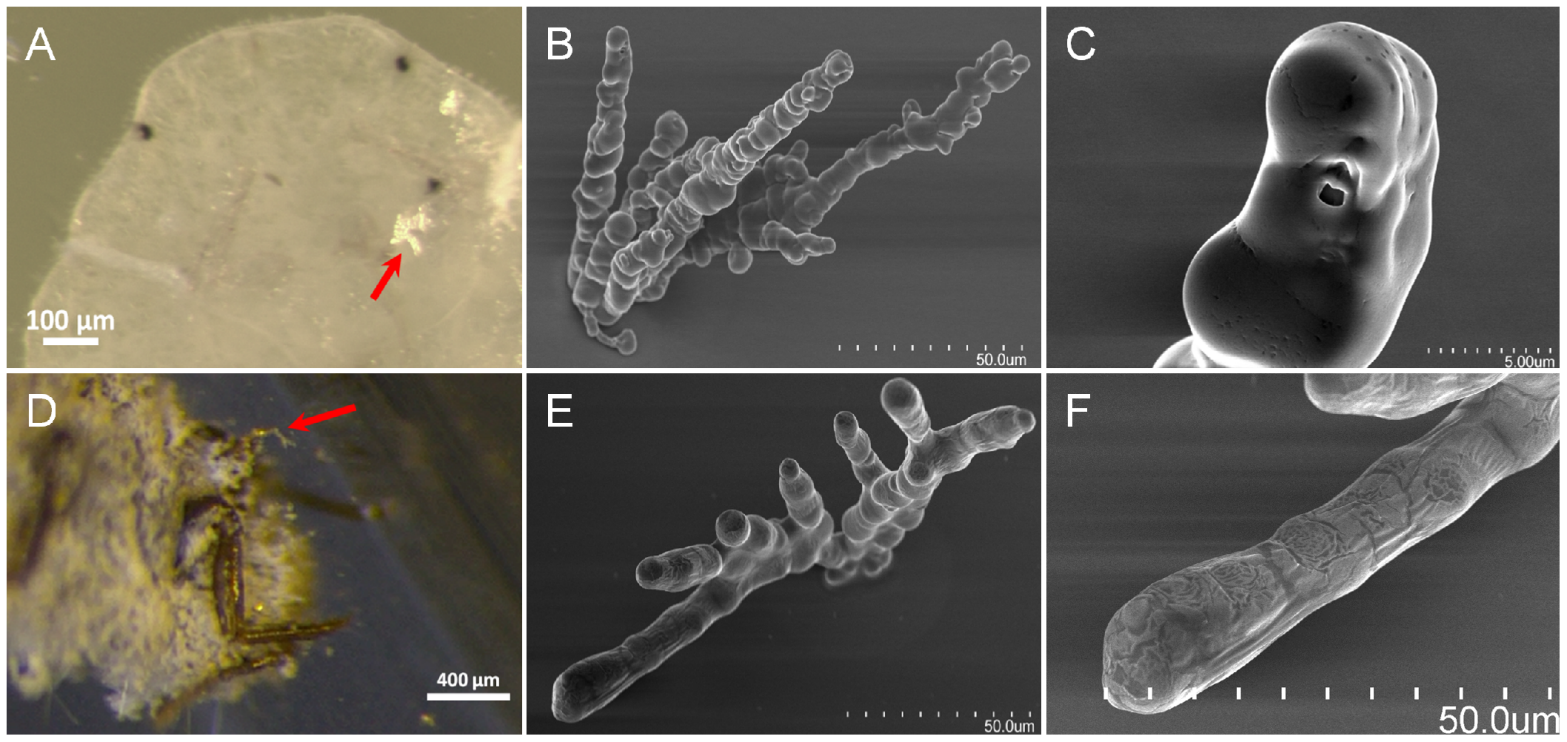
*P.pyriformis* and *F. virgata in āere* formed stylet sheaths. Panels A – C and D – F are *P. pyriformis* and stripped *F. virgata* associated micrographs, respectively. Panels A and D are stereoscopic views of a *P. pyriformis* and *F. virgata* (respectively) mock feeding across a clear plastic membrane, with red arrows indicating multi-branched air formed sheaths. Panels B and E are SEM micrographs of *P. pyriformis* and *F. virgata* multi-branched sheaths, respectively. Panels C and F are magnified SEM micrographs of selected terminal sheath branch tips from Panels B and E, respectively.

**Figure 4:**
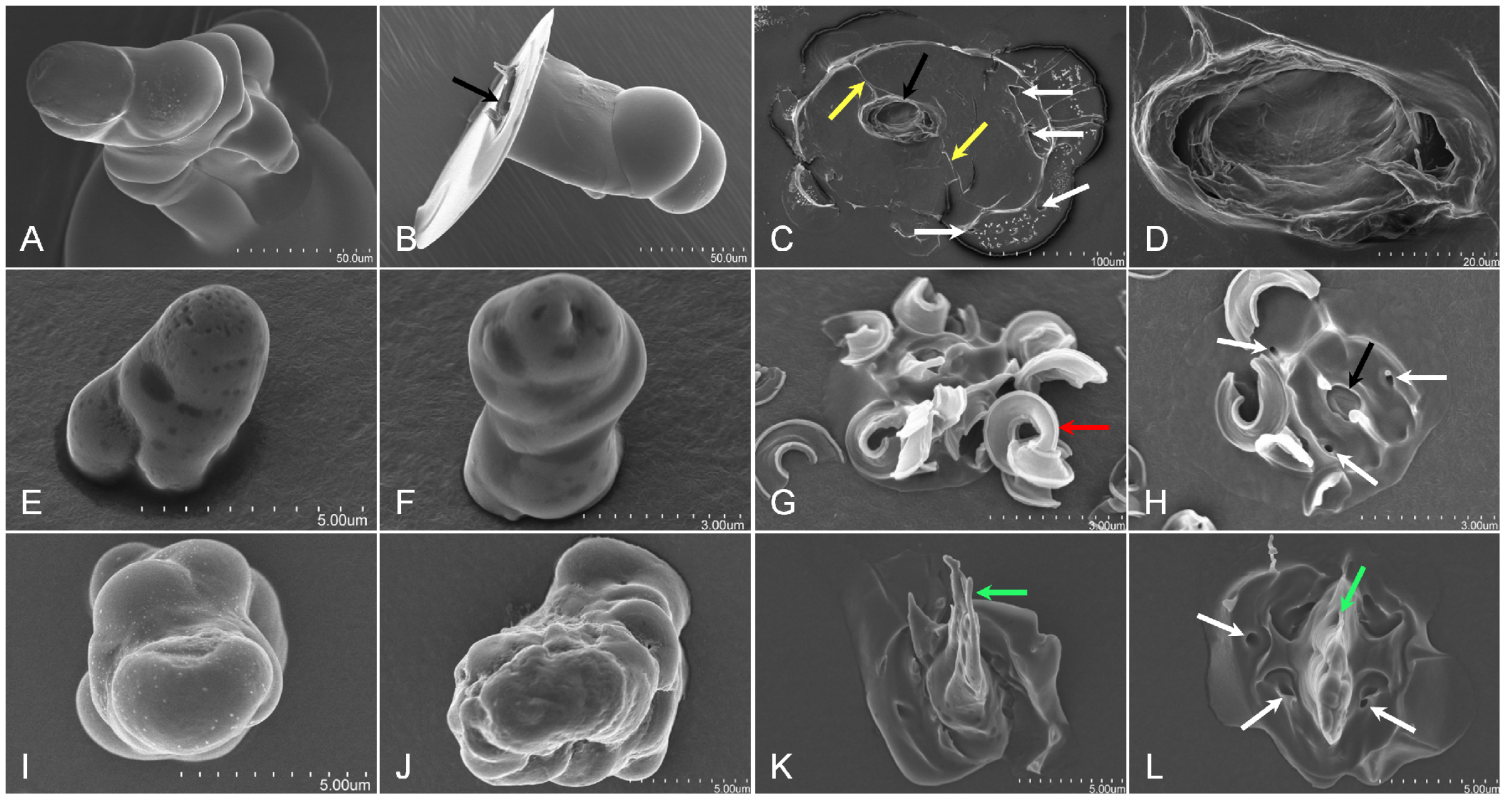
*H.vitripennis*, *B. tabaci*, and *A. nerii in āere* formed sheaths and flanges. Panels A – D, E – H, and I – L are sheath and flanges from *H. vitripennis*, *B. tabaci*, and *A. nerii*, respectively. Panels A, B, E, F, I, and J are sheath and Panels C, D, G, H, K, and L are flange SEMs, respectively. For each, the sheaths are short and lack the branching as seen previously for *D. citri*, *P. pyriformis*, and *F. virgata* (for comparison see Figure 1 - Panels A and B, Figure 2 - Panel D composite, and Figure 4 - Panels B and E). Flanges for both the *H. vitripennis* (Panel C) and *B. tabaci* (Panel H) indicate an open access point into the central canal (designated by the black arrows) for the stylets. Panel D is an increased magnification SEM of the *H. vitripennis* flange opening for the stylets indicating the hollow canal for the stylets. Panels K and L indicate that the *A. nerii* flange are sealed closed by retraction secreted sheath material (green arrows). White arrows indicate sensilla cavities in the flange surface (Panels C, H and L), yellow arrows indicate the labial groove imprint on the *H. vitripennis* flange surface (Panel C), the red arrow indicating the coalescence of secreted whitefly ‘waxy’ cuticular lipid hydrocarbons shed and covering the flange (Panel G), and the black arrows (Panels B and H) indicate the open cavity of the stylet canal for both the *H. vitripennis* and *B. tabaci* flanges.

To determine if *in āere* stylet sheath production was an attribute of other phytophagous hemipterans, similar MFCs were tested with: *P. pyriformis* (Figure 3A–C), *F. virgata* (Figure 3D–E), *H. vitripennis* (Figure 4A and B), *B. tabaci* (Figure 4E and F), and aphid (Figure 4I and J) insects with each of these produced *in āere* stylet sheaths (see also Video’s S2– S6). Observations indicated that *D. citri*, *F. virgata*, and *P. pyriformis in āere* sheaths were longer and more complex (including commonly multi-branched stylet sheaths being formed) than those deposited by aphids, *B. tabaci*, and *H. vitripennis* (compare Video’s S1, S5, and S6 to Video’s S2, S3, and S4) under this artificial system. Penetration of the stylet bundle and formation of the stylet sheath for each of these insects typically occurred within the first 0–10 minutes post caging in the MFCs, with extended incubation times (beyond 10 minutes and up to 72 hours post caging in the MFC) providing an increase of deposited stylet sheaths for *D. citri*, aphids, and WFs (data not shown).

To gauge the proclivity of singularly caged *D. citri* (in a MFC) to form stylet sheaths *in āere* versus ‘in diet’ over a 24 hours period, we found these averaged ∼16.2±2.1 stylet sheaths *in āere* relative to 53.0±9.6 (mean±standard error, n = 6) for singularly caged ‘in diet’ fed psyllids.

Contrastingly, it was observed that singularly MFC caged *H. vitripennis* produced approximately 2–7 sheaths during the first ten minutes after caging; however, subsequently the *H. vitripennis* ceased probing over the following 24-hour period. For *F. virgata* and *P. pyriformis,* these were observed to produce complex multi-branched (Figure 3) *in āere* stylet sheaths with continued probing attempts over the 24-hour period; however, the total number of sheaths produced by these remained low, perhaps due to the sedentary nature of these or the complexity of their stylet sheaths formed in the MFC environment.

### Direct Surface Microscopy (SEM, Stereo, and Light)

The MFCs (see methods) proved to be an adaptable platform to allow for new observational methods by SEM and light microscopy to obtain both high resolution still and video images of form and formation of stylet sheaths for these insects. For direct surface SEM both the sheath face (Figure 3G and E; Figure 4A and B, E and F, I and J; and Video’s S1, S2, S3, and S6) and the flange face (Figure 2H; Figure 4C and D, G and H, K and L; Figure S1A–F; and Video S1) of the MFC membranes were observed. Stereomicroscopy (Figure 3A and D; and Video’s S1, S2, S4, S5 and S6) and inverted light microscopy (Video’s S1, S3, and S5) of MFC chambers provided both still and video imagery of stylet sheath formation without solid or liquid diet obstruction.

### Descriptions of *in āere* Stylet Sheaths of Tested Insects

#### Diaphorina citri

**Figure 5:**
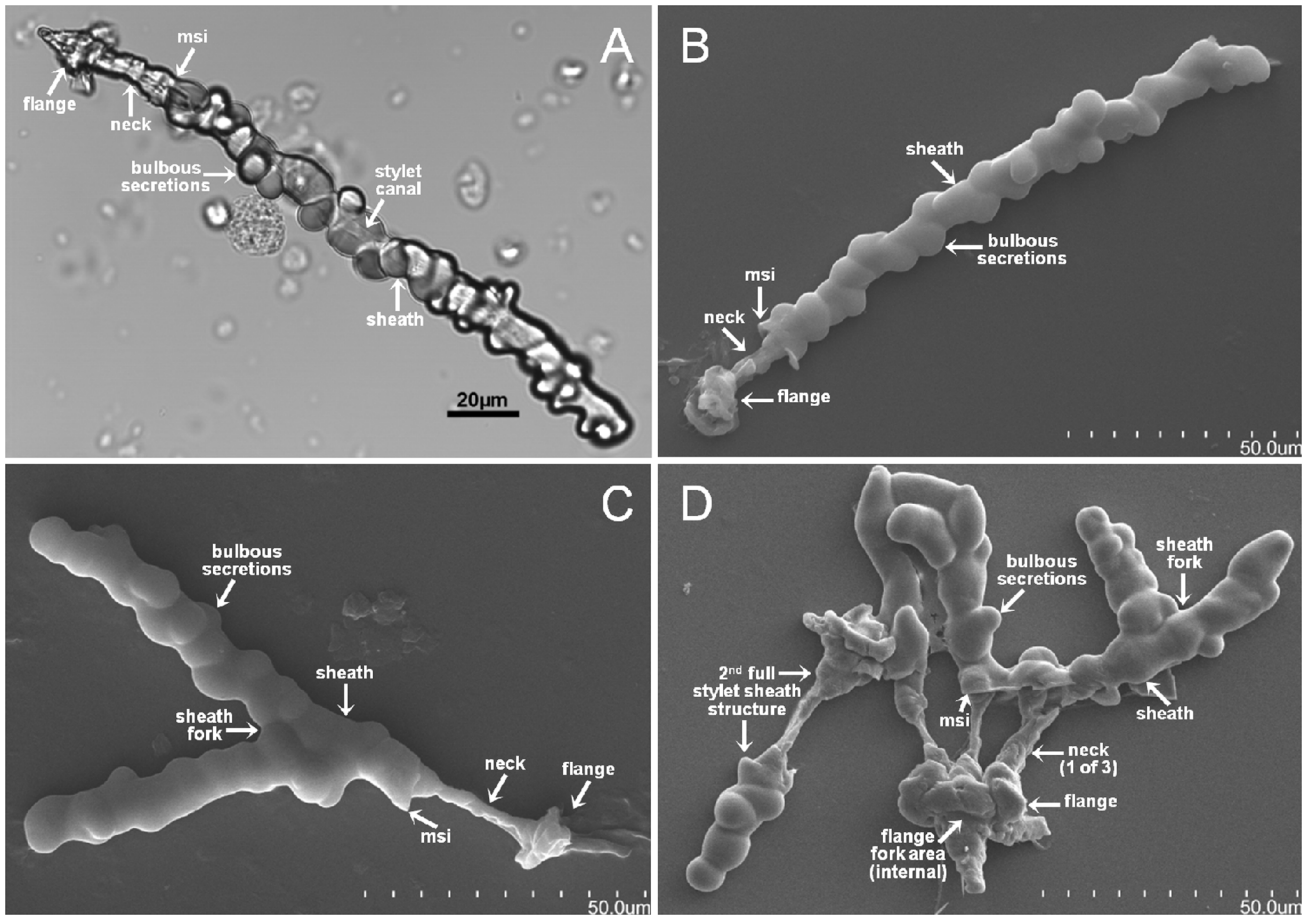
*D.citri in a¯ere* formed stylet sheaths from water-soluble Solvy™ stabilizer membranes. Panel A image is DIC and panels B – D are SEM micrographs of *D. citri* Solvy™ isolated stylet sheaths. Structural features of these intact (attached sheath with flange) stylet sheaths are indicated including: ‘neck’ segments that correspond to the membrane traversing span connecting the flange and sheath segments (Panels A – D), the membrane sheath interface (msi) on the sheath face side (Panels A – D), continuous bulbous secretion formations and fork locations in the sheath segment (Panels C and D), or the flange (Panel D) segment.

**Figure 6:**
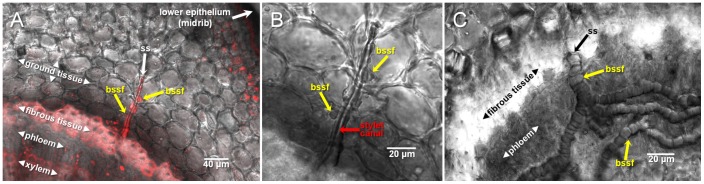
Naturally formed *D.citri* stylet sheaths within leaf midrib of Duncan grapefruit (*Citrus paradisi* Macfadyen). Panel A indicates a stylet sheath segment having bulbous stylet sheath formations (bssf) within the ground tissue region of the midrib that originated from a *D. citri* probe on the lower surface of the midrib. Propidium iodide fluorescence staining is used to provide contrasting resolution between the tissue types and the stylet sheath structure. Panel B is a magnified view of Panel A stylet sheath (ss) region minus fluorescence, indicating the bssf areas as well as the apparent stylet canal traveling parallel to the stylet sheath core. Panel C illustrates bssf continuing within the phloem tissue and additionally illustrates the multi-branching of the sheath portion within the phloem tissue. Micrographs are DIC.

Characteristic attributes of *in āere D. citri* stylet sheaths include: a sealed flange (Figure 2H, Figure S1A; and Video S1), labial sensilla impression cavities in the flange surface (Figure 2H and Figure S1A), neck region (Figure 5A–D; Figure S1G–I; and Video S1), and bulbous secretions on the sheath segment of the stylet sheath (Figure 1A, E–H; Figure 2D, Figure 5A – D; Figure S1G–I; and Video S1). *D. citri in āere* stylet sheaths frequently included multiple branches (Figure 1A; Figure 5C and D; Figure 6C; Figure S1I; and Video S1). These multi-branched stylet sheaths showed that branch forks can occur within the flange (Figure 5D and Figure S1I) or within a sheath segment (Figure 1A; Figure 5C and D; and Video S1). This is consistent with *in planta D. citri* stylet sheaths, Figure 6, which show similar traits of bulbous secretions (panels A and B) and multiple branching points (panel C). The branching in this instance occurs within the phloem region of the leaf tissue (Figure 6C).

Additional *in āere* sheath morphologies are illustrated by the *D. citri* stylet bundles ability to angularly probe as Figure 1E contains a single track stylet sheath having an ∼93° bend central to the sheath. SEM and light microscopy illustrate that the central canals are generally hollow through the sheath core (Figure 1B–D; Figure 2A–D; Figure 5A; Figure S1G–H; and Video S1), having an average canal diameter of ∼2.5 to 3.5 µm diameter for adult *D. citri* stylet sheaths; however, Figure S1G–H (with insets) illustrate these canals can be both hollow and filled-in/obstructed at points along the central canal track. Video S1 illustrates that the hardening of the *D. citri in āere* stylet sheath material appears to occur in as few as 45 seconds upon first detection of the secretion of saliva from the stylet bundle post piercing of the MFC membrane. Individual MFC probing events by *D. citri* can be highly variable, ranging from a few seconds, to a few minutes, and can extend beyond ten minutes (Video S1 and observations).

#### Aphis nerii, toxoptera citricida, aphis gossypii

Characteristic attributes of *in āere* aphid stylet sheaths indicate sealed flanges are common across these aphid genera (Figure 4K and L; Figure S1B). *A. nerii* (Figure 4I and J) and *T. citricida* (data not shown) commonly have diminutive sheaths; however, *A. gossypii* illustrates the potential to make multi-branch stylet sheaths (Video S2) *in āere*. Bulbous secretions are apparent on the stylet sheath (Figure 4I and J) and the flange displays the presence of labial sensilla cavity impressions (Figure 4L; and Figure S1B).

#### *Bemisia tabaci* biotype B

*B. tabaci in āere* stylet sheaths were commonly short, most often having a single canal (Figure 4E and F; Video S3), but occasionally with multiple canals (Video S3). In general, bulbous secretions constitute the structural morphology of these short sheath structures (Figure 4E and F; and Video S3). *B. tabaci in āere* formed flanges remain open to the stylet canal (Figure 4H), and labial sensilla impression cavities are evident in the flange (Figure 4H). Secreted whitefly ‘waxy’ cuticular lipid hydrocarbons [35], [36], [37] are prominently shed across the surface of the flange membrane face, coalescing in high numbers on the *B. tabaci* flange (Figure 4G and H; and Figure S1C), often obstructing the view of the flange.

#### Homalodisca vitripennis

Attributes of *in āere H. vitripennis* stylet sheaths appear as semi-bulbous to somewhat vertical amorphous protrusions from the sheath face of the MFC membrane (Figure 4A and B; and Video S4). Relative to the size of this insect and its stylet bundle, their stylet sheaths appear as short, single canal structures, and do not indicate multi-branching *in āere* (Figure 4A and B; and Video S4). The solidification rate of *H. vitripennis in āere* stylet sheaths may differ relative to the probing membrane used for the MFC as clear plastic wrap and parafilm surfaces seem to indicate a difference in rigidity upon sheath retraction of the *H. vitripennis* (Video S4). The *H. vitripennis* flanges indicate an open central canal of the stylet sheath (Figure 4C and D; and Figure S1D–F) with sensilla impressions in the flange surface and the labial groove imprints apparent (Figure 4C; and Figure S1D).

#### Ferrisia virgate

Characteristics of *in āere F. virgata* stylet sheaths appear almost exclusively as multi-branched structures having a continuous bulbous structural morphology from the sheath base. Branching is usually apparent along the main ‘trunk’ portion of the sheath protrusion away from the MFC membrane surface akin to branches from a tree (Figure 3D and E and Video S5). The branches appear to exist on the same plane and do not form a radial pattern from the branching point (Figure 3E and Video S5). *F. virgata in āere* flanges were not studied for this report. Behavioral movements of the *F. virgata* during stylet sheath production *in āere* indicate both branching and radial movements of the sheath encased stylet bundle and a ‘push-up’ like action is employed by the *F. virgata* that appears to assist in the partial retraction of the stylet bundle to a branching point within the stylet sheath (Video S5). *F. virgata in āere* complete sheath formation time is a prolonged event that can last up to 17 minutes for a single (multi-branched) stylet sheath (Video S5).

#### Protopulvinaria pyriformis

Characteristic attributes of *in āere P. pyriformis* stylet sheaths appear almost exclusively to be multi-branched structures, having a continuous bulbous structural morphology originating from the stylet sheath base through to the tip of the sheath structure (Figure 3B and C; and Video S6). No evidence for single canal *P. pyriformis* stylet sheaths was observed. Branching appears to occur in a radial pattern from the sheath base near the MFC membrane surface (Figure 3A and B; and Video S6). This *P. pyriformis* basal branching appears similar to certain *D. citri* stylet sheaths that contained branching points located within the flange region (Figure 5D; and Figure S1I). *P. pyriformis* flanges were not studied for this report. Time for sheath production does not appear to be a comparable feature as the *P. pyriformis* probed in a single location from which it did not appear to move during a 24-hour observational period.

### Using Solvy™ Water-soluble Membrane for *D. citri* Stylet Sheath Collection

To collect full *D. citri* stylet sheaths for structural analyses two methods were used. Initially, we used a bent glass rod in combination with a small volume of water applied directly to the MFC membrane surface opposite the surface on which the psyllids fed. The bent glass rod was used to scour the MFC surface to dislodge *D. citri* stylet sheaths, which were then collected in the water/stylet slurry for analyses including microscopic observations (Figure 1 and Figure 2A–D). Less than 5% of the available stylet sheaths were recovered from either clear plastic wrap or parafilm membranes using this method. To increase the efficiency of sheath harvest, Solvy™ stabilizer (a water-soluble polyvinyl alcohol film) was used as the probing membrane for MFCs. This was used in combination with filters (see methods) to harvest (conservatively) an estimated >75% of the available stylet sheaths. This technique also allowed the harvest of intact stylet sheaths including the flange, neck, and sheath portions (Figure 5). In contrast, only the sheath portion was harvested by the bent glass rod/scour method (Figure 1 and Figure 2A–D).

## Discussion

The formation of *in āere* stylet sheaths by *D. citri*, *A. nerii*, *A. gossypii*, *T. citricida*, *B. tabaci*, *H. vitripennis*, *F. virgata*, and *P. pyriformis* illustrates formation of sheaths without host tissue (plant) or non-host liquid or agar medium interactions or stimuli. Additional findings from this study include: ***i***) stylet sheath formation does not require a persistent resistance against the stylets (post penetration of the membrane) as a stimulation to elicit sheath secretions as previously suggested [19]; as the stylet bundle penetrates through the membrane material, air resistance is minimal, and sheath formation still occurs (Video’s S1– S6); ***ii***) stylet probing of the membrane was virtually instantaneous upon caging in the MFCs for each of these phytophagous hemipterans; ***iii***) the insects did not require an additional stimuli to induce these probing events, suggesting a proclivity for exploratory probing of their environment; and ***iv***) solidification of stylet sheaths appeared to occur rapidly *in āere*. These findings expand our knowledge of stylet sheath formation for these tested hemipterans; however, they do not indicate whether molecular compositional differences exist between *in āere* versus *in planta* formed stylet sheaths.

Common similarities in physical/morphological form appear between both *in āere* (Figure 1, 2, 3, 5 and Figure S1) and *in planta* (Figure 6) *D. citri* stylet sheaths, including flange, sheath branching, bulbous formations, and neck regions. These similarities between these *D. citri in āere* and *in planta* sheath forms may suggest a common chemical/structural composition as the mechanisms for sheath deposition may be similar; however, comparative chemical analyses of these should clarify this. Interestingly, a comparison of the neck regions from Solvy™ isolated *D. citri* stylet sheaths (Figure 5A–D) appears to correlate with previous reported descriptions of *in planta* sheaths from other hemipterans: the psyllid *Ctenarytaina spatulata* (Brennan *et al* 2000 Figure 2B [27]) and the planthopper *Nilaparvata lugens* (Wang *et al* 2008 Figure 6A, B, and D [22]). In these descriptions, the neck regions are indicated *in planta* as the stylet sheaths pass through the upper epidermal layer of the host plant tissue.

Common morphological features of *in āere* and *in planta* stylet sheaths support the use of ‘mock feeding chambers’ as a viable means for behavioral and stylet sheath compositional analyses for these hemipterans. Here we demonstrate the flexibility of MFC systems by altering the probing membrane types for specific purposes. For instance, the use of clear plastic wrap allowed for highly magnified real-time microscopic observations and live action video of hemipteran stylet sheath formation (e.g. Video S3). The use of Solvy™ water soluble membrane provided the means to isolate intact sheaths providing for in-depth compositional analyses (Morgan, J. K., *et al*, unpublished data) that lack potentially confounding background elements (traces of artificial diet or host plant tissue). Therefore, varying the membrane type for different MFC configurations may be useful for alternate analytical purposes for hemipteran stylet sheath or related studies.

Our results also present a method from which environmental influences can affect behavioral activities. For example, *H. vitripennis*, *B. tabaci*, and aphids each produce short sheaths *in āere* (Figure 4A and B, E and F, I and J); however, previous literature indicates that stylet sheaths for *H. vitripennis*, *B. tabaci*, and aphids have long and/or multi-branched sheaths *in planta* [31], [38], [39]. Therefore, manipulations of chemical applications to either the flange or sheath faces of an MFC membrane could provide a method to monitored for influences on the feeding behavior in relation to sheath structure and length. This common attribute of short sheaths produced on MFCs by *H. vitripennis*, *B. tabaci*, and aphids is most likely a manifestation of these insects ability to ‘sense’ the environment on which it is feeding and ‘deciding’ that the environment represents an ‘unsuitable’ feeding site; hence, these spend less energy by reducing their probing efforts. Clearly, the insects have different responses in this process since the MFC naïve *H. vitripennis* probe initially often in rapid succession ∼2 to 7 times upon caging (typically within the first ten minutes) and then stop while naïve *D. citri* probe continually throughout a 24-hours (and beyond) averaging approximately 16 (±2.1 St. Error) probes per 24-hour period. The membrane feeding system we present in this paper should be readily adaptable for further studies on this behavior.

Alternate differences are evident when comparing the flange regions of stylet sheaths of *D. citri* and aphids versus *H. vitripennis* and *B. tabaci* (we did not examine the flanges for *F. virgata* and *P. pyriformis*). For *D. citri* (Figure 2H, Figure S1A) and aphids (Figure 4K and L, Figure S1B), micrographs indicate closure of the flange openings. In contrast, those of *H. vitripennis* (Figure 4C and D, Figure S1 D–F) and *B. tabaci* (Figure 4H) indicate the flange central canal remains open upon stylet retraction. Present literature also indicates open flanges are evident on host leaf surfaces fed on by glassy-winged sharpshooter (Leopold *et al* 2003 Figure 4C and D, Figure S1D–F, Figure 28 [31]) and whiteflies (Freeman *et al* 2001 Figure 4H, Figure 1 [39]), which is consistent with our *in āere* flange findings for these insects. These data support a hypothesis that *D. citri* and aphids secrete sheath material during retraction of the stylet bundle resulting in flange closure.

For *D. citri*, supplemental evidence supporting sheath material secretion upon stylet bundle retraction is indicated by the variable diameters within individual canals for *D. citri* stylet canal tracks that appear to range from ∼2.5 ∼3.5 µm (Figure 2D, Figure 5A, and Figure S1G–I). Evidence that stylet canals can be completely filled for *D. citri* (Figure S1G–I, inset images) also strongly indicate this. As the average adult *D. citri* stylet bundle diameter is ∼5 µm (Figure 2F, note flange circumambient the stylet bundle indicating that the complete stylet bundle enters the canal of the stylet sheath), a *D. citri* stylet sheath canal having a diameter less than 5 µm may suggest that stylet bundle retraction secretion is involved or that drying effects of the stylet sheath have constricted the canal diameter [10].

The mechanism for the solidification of stylet sheath has been thought to require interaction with host plant tissues [20] or artificial media, yet these *in āere* stylet sheaths refute this for these tested Hemipterans (Figure 1, 2, 3, 4, 5, and Video’s S1, S2, S3, S4, S5, S6). As referenced previously, prior work has implied *in āere* solidified sheath material occurring in sharpshooters [29]. In this prior study, the authors indicate specific protein differences between the profiles of brush induced sheath materials and sheath materials harvested by differing methods (parafilm collected over diet, direct gland removal saliva harvest, and filter paper extracts ‘milking’). Their data indicated the necessity of protein factors for full stylet sheath formation to occur [29]. Other hypotheses suggest that oxygen specific interactions are needed for proper sheath gelling [11] and support for this has recently been indicated for aphid stylet sheaths [10]. Experiments using the *in āere* system shown here can be envisioned where the gas composition of the feeding chamber can be manipulated to block oxygen from below the membrane surface. This would provide a convenient alternate method of testing oxygen requirements.

This study demonstrates that the ability to form stylet sheaths *in āere* is a common trait for multiple taxonomically diverse hemipterans (*D. citri*, *A. nerii*, *A. gossypii*, *T. citricida*, *B. tabaci*, *H. vitripennis*, *F. virgata*, and *P. pyriformis*). Each probed the membrane of their MFCs regardless of membrane type within the first few minutes of caging. Differences in stylet sheath lengths by species on MFCs solicit further inquiry into potential insect specific sensory capacities. We also demonstrated the adaptability of MFC membranes for alternate analyses related to stylet sheath production. The water-soluble Solvy™ stabilizer MFC membranes provides a simple way to rapidly isolate intact stylet sheaths to perform a variety of chemical and other analyses. Further work is ongoing, but these techniques pave the way for additional rapid advancements in the study of hemipteran stylet sheaths biosynthesis and potential ways to combat these pests by molecular interventions that target the stylet sheaths.

## Supporting Information

Figure S1 **Micrographs of** ***in āere*** **formed flanges and stylet sheaths from:** ***D. citri*** **,** ***T. citricida*** **,** ***B. tabaci*** **, and** ***H. vitripennis*** **.** Panels A and B are SEM micrographs of *D. citri* and *T. citricida* flanges (respectively), with green arrows indicating retraction secreted sheath (rss) material and white arrows indicating flange sensilla cavities (ssc). Panel C is a SEM micrograph of a typical *B. tabaci* flange coated with a heavy coalescence of the *B. tabaci* ‘waxy’ lipid hydrocarbon secretion (red arrow). Panel D is a SEM micrograph overview of a *H. vitripennis* flange with the black arrow indicating the open cavity entrance for the stylet canal, yellow arrow indicating the labial groove imprint on the flange surface, and the white arrows indicating *H. vitripennis* ssc imprints. Panel E is a higher magnification SEM micrograph surface focused view of the *H. vitripennis* flange stylet canal opening from Panel D, with Panel F being a deeper inside view focus SEM micrograph of the Panel D stylet canal opening. Panels G – I (including enlargement inset images), of full *D. citri* stylet sheaths (from Solvy™) indicating stylet canal closure throughout the central portion of the sheath (black arrows).
(TIF)

Video S1 **Video of a** ***D. citri*** **(Hemiptera: Psyllidae, Asian citrus psyllid) forming an** ***in āere*** **stylet sheath.** From approximately 1 minute to 1 minute 45 seconds of the video, stylet sheath material appears to solidify. Additionally, scanning electron micrographs of both single canal and multi-branched sheaths and a flange are indicated, as well as a light microscopy of a fully formed Solvy isolated ss (flange and sheath connected by a neck region).
(MP4)

Video S2 **Video segments of two individual** ***A. gossypii*** **(Hemiptera: Aphididae, cotton/melon aphid) forming** ***in āere*** **stylet sheaths.** Additionally, scanning electron micrograph indicates a multi-branch sheath formed *in āere*.
(MP4)

Video S3 **Video of a** ***B. tabaci*** **biotype B (Hemiptera: Aleyrodidae, whitefly) forming an** ***in āere*** **stylet sheath.** Additionally observable are copious cuticular secretions on the flange face of the plastic wrap (appearing as white dust like particles). With scanning electron micrographs that indicate additional *B. tabaci* short stylet sheaths formed *in āere*.
(MP4)

Video S4 **Three video segments of** ***Homalodisca vitripennis*** **(Hemiptera: Cicadellidae, glassy-winged sharpshooter) forming** ***in āere*** **stylet sheaths.** Video segments 1 and 2 indicate stylet sheath formation *in āere* across clear plastic wrap. Video segment 3 indicates stylet sheath formation *in āere* across a Parafilm® membrane.
(MP4)

Video S5 **Video of a single** ***Ferrisia virgata*** **(Hemiptera: Pseudococcidae, striped mealybug) forming a multi-branch stylet sheath** ***in āere*** **.** Total elapsed time from start to finish is approximately 17 minutes. Subsequent light micrographs show a detailed view of the sheath.
(MP4)

Video S6 **Video of a** ***Protopulvinaria pyriformis*** **(Hemiptera: Coccidae, pyriform scale) forming a multi-branched** ***in āere*** **stylet sheath.** Primary and secondary segments indicate dorsal and ventral stereo micrograph images of a *Protopulvinaria pyriformis* (Hemiptera: Coccidae, pyriform scale) on a leaf and caged in a mock feeding chamber forming a stylet sheath (respectively). Subsequent to these, a stereoscopic video of a single scale indicates the formation of a multi-branch stylet sheath across the clear plastic wrap of the mock feeding chamber. The final segment indicates a scanning electron micrograph of the multi-branch stylet sheath formed from the pyriform scale of the video.
(MP4)
