# Supplementary figures and images for: Formation of Stylet Sheaths in āere (in air) from Eight Species of Phytophagous Hemipterans from Six Families (Suborders: Auchenorrhyncha and Sternorrhyncha)
Source: PLoS One. 2013 Apr 24;8(4):e62444. doi: 10.1371/journal.pone.0062444 (PMC3634779; doi:10.1371/journal.pone.0062444)

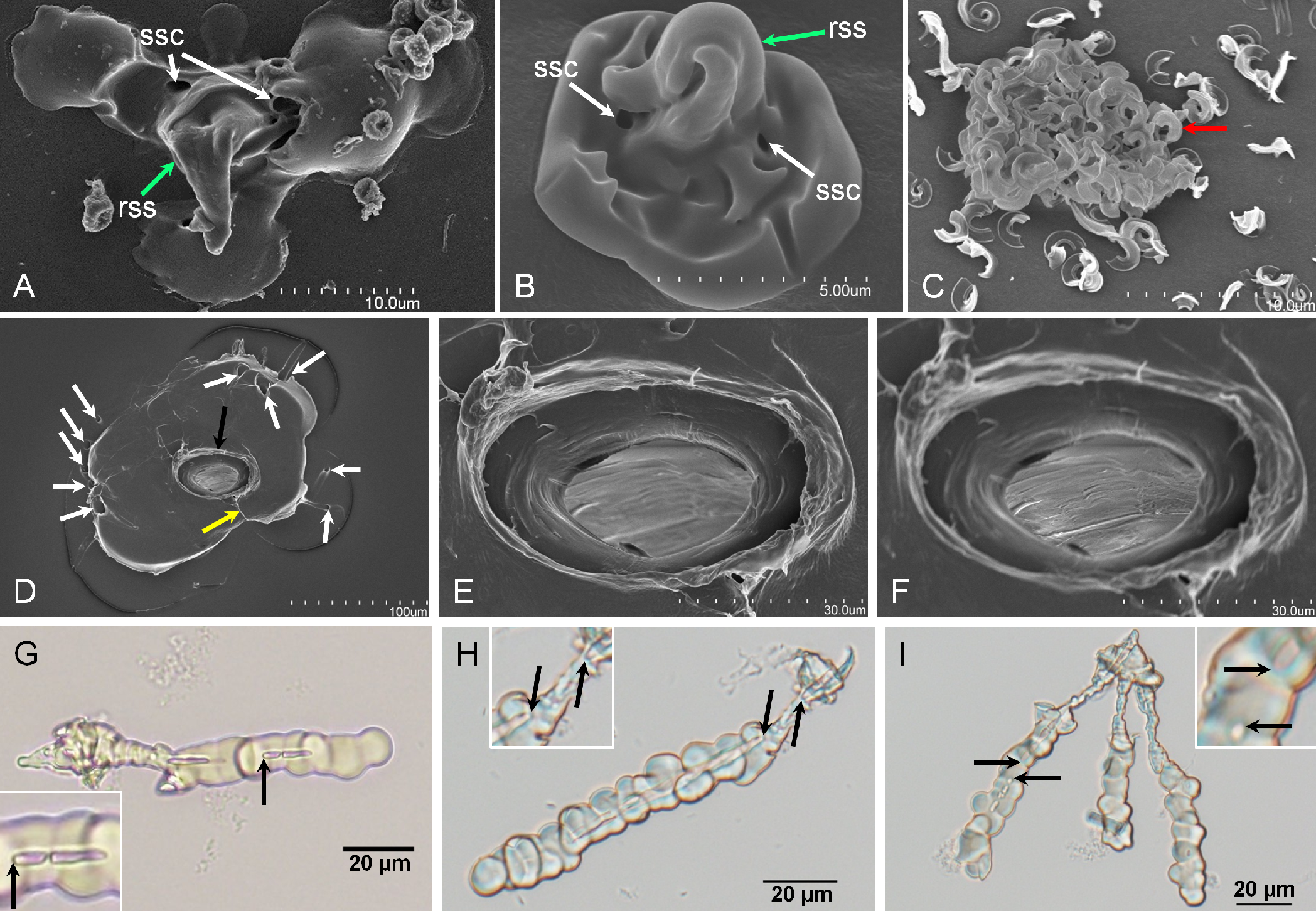

Supplement: Figure S1 — Micrographs of in āere formed flanges and stylet sheaths from: D. citri , T. citricida , B. tabaci , and H. vitripennis . Panels A and B are SEM micrographs of D. citri and T. citricida flanges (respectively), with green arrows indicating retraction secreted sheath (rss) material and white arrows indicating flange sensilla cavities (ssc). Panel C is a SEM micrograph of a typical B. tabaci flange coated with a heavy coalescence of the B. tabaci ‘waxy’ lipid hydrocarbon secretion (red arrow). Panel D is a SEM micrograph overview of a H. vitripennis flange with the black arrow indicating the open cavity entrance for the stylet canal, yellow arrow indicating the labial groove imprint on the flange surface, and the white arrows indicating H. vitripennis ssc imprints. Panel E is a higher magnification SEM micrograph surface focused view of the H. vitripennis flange stylet canal opening from Panel D, with Panel F being a deeper inside view focus SEM micrograph of the Panel D stylet canal opening. Panels G – I (including enlargement inset images), of full D. citri stylet sheaths (from Solvy™) indicating stylet canal closure throughout the central portion of the sheath (black arrows). (TIF) [file pone.0062444.s001.tif]
